# StemOne^TM^/Stempeucel^®^: CDSCO Approved, Adult Human Bone Marrow-Derived, Cultured, Pooled, Allogenic Mesenchymal Stem Cells for Knee Osteoarthritis

**DOI:** 10.3390/biomedicines11112894

**Published:** 2023-10-26

**Authors:** Ashim Gupta

**Affiliations:** 1 Regenerative Orthopaedics, Noida 201301, India; ashim6786@gmail.com; 2 Future Biologics, Lawrenceville, GA 30043, USA

The knee, the most likely joint to present osteoarthritis (OA), is accountable for approximately 80% of the global burden of the OA [1,2]. The prevalence of knee OA has continued to increase over the last two decades, with no indications of slowing down [1,2]. Its etiology entails inflammation of the synovial tissue and degeneration of the articular cartilage, resulting in unendurable pain and loss of function [3,4]. Conservatively, knee OA is managed by using pharmacological agents including non-steroidal anti-inflammatory drugs (NSAIDs), narcotics, viscosupplementation, and corticosteroids; non-pharmacological modalities including weight loss, diet change, activity modification, and physical therapy; and surgery (particularly in later stages of knee OA), when the conventional treatment options have been unsuccessful [3,4]. These above-mentioned treatment modalities have shortcomings and side effects, consistently aiming to decrease pain instead of targeting the underlying pathophysiology [3,4].

Lately, several molecular targets, including interleukin-1 (IL-1), transforming growth factor-β (TGF-β), matrix metalloproteinases (MMPs), etc., have been reported to be associated with the etiopathogenesis of OA [5,6,7]; nevertheless, numerous treatments may well have a negative risk-to-benefit ratio [8,9]. Subsequently, further safe and effective treatment alternatives will be necessary if we are to manage this unmet medical necessity.

Recently, there has been an increased interest in the use of biologics for regenerative medicine applications, specifically for musculoskeletal conditions, including knee OA [1,2,3,4,10,11,12]. Among several biologics, the use of autologous or allogenic mesenchymal stem/stromal cells (MSCs) have quickly emerged for management of knee OA [13,14,15]. The increased interest in the use of MSCs can be attributed to their anti-inflammatory and immunomodulatory properties; their ability to secrete bioactive molecules such a, growth factors, cytokines, and extracellular vesicles, including exosomes and extracellular matrix (ECM) components; and their potential to repair cartilage via their ability to differentiate into chondrocytes, induce proliferation and maturation of healthy chondrocytes, and induce differentiation of chondroprogenitor cells [16]. Several studies have reported that the administration of autologous MSCs is safe and effective in knee OA patients [17,18,19]; however, there is insufficient literature assessing the safety and efficacy of allogenic MSCs.

In this editorial, I focus on the clinical outcomes of two studies, a Phase II and a Phase III clinical trial by Gupta et al. [20,21], entitled, “Efficacy and safety of adult human bone marrow-derived, cultured, pooled, allogeneic mesenchymal stromal cells (Stempeucel^®^): preclinical and clinical trial in osteoarthritis of the knee joint” and “Efficacy and Safety of Stempeucel in Osteoarthritis of the Knee: A Phase 3 Randomized, Double-Blind, Multicenter, Placebo-Controlled Study”, where the authors investigated the safety and efficacy of adult human bone marrow-derived, ex vivo-cultured and expanded, pooled, allogeneic MSCs (**Stempeucel^®^**, Stempeutics Research Pvt Ltd., Bangalore, India; currently distributed by Alkem Laboratories in India as **StemOne^TM^**; **approved by the Central Drugs Standard Control Organisation** **(CDSCO)**, New Delhi, India) for the management of symptomatic knee OA.

In the first study, a multi-center, randomized, double-blinded, placebo-controlled, Phase II dose-finding clinical study, Gupta et al. [20] investigated the safety, potential efficacy, and appropriate dose of intra-articular administration of these allogenic bone marrow-derived MSCs (BMSCs) in knee OA patients. The key inclusion criteria included patients in the age range of 40–70 years; radiographic evidence of Grade II or III (on Kellgren-Lawrence scale); history of primary idiopathic OA of the knee characterized by pain requiring intake of analgesics; self-reported difficulty in one of the following activities: lifting and carrying groceries, walking 400 m, getting in and out of a chair, or going up and down stairs; and on stable medication including NSAIDs, opioids, or opiates for the last 3 months. The main exclusion criteria included prior or ongoing medical conditions that can affect the safety of subjects; history of surgery or major trauma to the study joint; arthroscopy on the study joint in the last 12 months; signs of active study joint inflammation and/or, if qualifying with OA of the knee, a large, bulging effusion of the study knee joint with the loss of normal contour of the joint at the screening visit or at the baseline examination; received intra-articular steroids or hyaluronan within the last 3 months; infections in or around the knee; awaiting a replacement knee or hip joint; other conditions that caused pain; deformity of the knee joint; significantly incapacitated or disabled; other known rheumatic or inflammatory disease; other pathologic lesions on X-rays of the knee; and infectious diseases or bleeding disorders. As many as 62 out of 82 patients screened met the inclusion/exclusion criteria, and 60 patients were enrolled (2 dropouts) in this study. Four different levels of dosages (25, 50, 75, and 150 million cells) were studied, and 15 patients in a 2:1 ratio (cells: placebo) were randomized at each dosage level. A total of 25 and 50 million cells dosages were suspended in 2 mL, and 75 and 150 million cells dosages were suspended in 4 mL of PlasmaLyte-A solution (mix of 5.26 g/L sodium chloride, 0.37 g/L potassium chloride, 0.30 g/L magnesium chloride hexahydrate, 3.68 g/L sodium acetate trihydrate, 5.02 g/L sodium gluconate, and sodium hydroxide (q.s. pH) in water for injection), respectively. For placebo control, the same volume of PlasmaLyte-A solution was used. A total of 30 min prior to the intra-articular injection of cells or placebo in the patellofemoral joint, 100 mg of hydrocortisone and 45.5 mg of pheniramine maleate were intravenously infused to prevent any potential anaphylactic reaction. After administration of cells or placebo, 2 mL of hyaluronic acid (Hyalgan, 20 mg/2 mL) was injected. Patients were assessed using the Visual Analogue Scale (VAS) for pain, and the Intermittent and Constant Osteoarthritis Pain (ICOAP) and Western Ontario and McMaster Universities Osteoarthritis (WOMAC-OA) indices for pain and function at baseline and at 1, 3, 6, and 12 months of follow-up. Radiographic analysis and magnetic resonance imaging (MRI) were carried out at baseline and at 6 and 12 months follow-up. MRI images were scored to assess the Whole-Organ Magnetic Resonance Imaging Score (WORMS). No adverse effects were reported related to the 25 million cells dosage, and nine mild-to-moderate adverse effects related to the 50, 75, and 150 million cells dosages and one severe adverse event related to the 150 million cells dosage were reported. The VAS score decreased over the study period for all groups except for the 150 million cells dosage, with maximum reduction in the 25 million cells group at 12 months follow-up compared to the other groups. A similar trend was observed for the WOMAC and ICOAP total and subscores. No clinically significant changes were observed on radiographic analysis or WORMS score. This study was not without limitations, including the small cohort size, which probably led to non-significant changes in the outcome measures compared to the placebo group, as well as unblinding at 6 months follow-up. Despite these shortcomings, this study is one of the first to demonstrate the safety and likely efficacy of ex vivo-cultured and expanded, pooled, allogenic BMSCs in patients with knee OA. This study also showed that the 25 million cells dosage is potentially the most effective among the tested dosages in terms of reducing pain and improving function. This laid the foundation for adequately powered, prospective, multi-center, double-blinded, randomized controlled trial to further evaluate the safety and efficacy of this dosage.

In the second study, a multi-center, randomized, double-blind, placebo-controlled, Phase III study, Gupta et al. [21] evaluated the safety and efficacy of intra-articularly administered 25 million aforementioned allogenic BMSCs for the management of symptomatic OA of the knee. The key inclusion criteria included patients aged 40–65 years; body mass index <30 kg/m^2^; history of primary knee OA; evidence of grade II to III OA (Kellgren-Lawrence scale); self-reported difficulty in one of the following activities: lifting and carrying groceries, walking 400 m, getting in and out of a chair, getting up from a squatting or cross-leg position, or going up and down the stairs; and use of analgesic medication for OA for 6 weeks based on the investigator’s judgment. The main exclusion criteria included subchondral sclerosis; MRI scan showing complete ACL or PCL tears, grade III meniscal tears, or exclusive patellofemoral arthritis; prior or ongoing medical conditions that can affect the safety of subjects; history of surgery or major trauma to the examined joint; arthroscopic surgery on the examined joint in the last 12 months; signs of active joint inflammation and/or large, bulging effusion with a loss of the normal contour of the joint at the screening visit or on the baseline examination; acute exacerbation of the examined joint in the past 6 weeks; use of intra-articular steroids or hyaluronan within the past 3 months; any stem cell treatment in the past by any route of administration; infection in or around the examined knee; awaiting replacement of the knee or hip joint; other conditions that can cause pain in the knee joint; gross deformity of the knee joint based on the principal investigator’s judgment; significantly incapacitated or disabled; any secondary causes of arthritis; and infectious diseases or bleeding disorders or allergy to hyaluronic acid. A total of 146 patients met the inclusion/exclusion criteria and were randomized via block randomization (block size of 4 patients each for Grade II and III) into two groups (allogenic BMSCs or placebo) with 73 patients/group. The patients in the allogenic BMSCs received 25 million cells suspended in 1 mL CryoStor CS5 (cryopreservation medium with 5% dimethyl sulfoxide (DMSO)) + 1 mL PlasmaLyte-A, followed by administration of hyaluronic acid (Hyalgan, 20 mg/2 mL). The placebo group received only 1 mL CryoStor CS5 + 1 mL PlasmaLyte-A, followed by administration of hyaluronic acid. Patients were assessed using WOMAC and VAS at 1 week and 1, 3, 6, and 12 months follow-up. Cartilage quality using T2 mapping and cartilage volume were also determined via MRI imaging at baseline and 6 and 12 months follow-up. The viability of cells was >90% and mean viable cell count was 26.5±0.9 million cells per vial. Mild-to-moderate adverse events. including injection site swelling and pain, were reported, though they subsided within few days. Allogenic BMSCs group showed significant improvement (*p* < 0.05) with respect to WOMAC total score and WOMAC pain, stiffness, and physical function subscores, as well as VAS score at both 6 and 12 months follow-up compared to the placebo control. T2 mapping showed no worsening of deep cartilage in the medial femorotibial compartment in the allogenic BMSCs group at 6 and 12 months follow-up. However, there was gradual worsening in the placebo group at 6 months and then to 12 months follow-up, which was statistically significant. Nonetheless, no significant differences were reported between the two groups. No changes were observed in the lateral femorotibial compartment for either group. Cartilage volume was higher in the allogenic BMSCs group compared to the placebo group, but the difference was not statistically significant. This study also has limitations, as also described by the authors, including the need to determine the number of optimal doses and their administration time-interval, as only a single dose was tested in this study. In spite of this, results demonstrated that the administration of these allogenic BMSCs is safe and effective in patients with Grade II or III knee OA.

In conclusion, despite the constraints, I applaud the efforts of the authors as they presented the medical community with much needed, well-executed, prospective clinical trials. These trials demonstrated that administration of ex vivo-cultured and expanded, pooled, allogenic BMSCs is safe and efficacious, in terms of reducing pain and improving function, in patients with Garde II or III knee OA. These trials also laid the foundation for adequately powered, multi-center, prospective, double-blinded, randomized controlled trials with longer follow-up duration, along with post-market studies, to further establish safety and effectiveness of this approved allogenic cell therapy.

## References

[1] Gupta A., Sharma S.P., Potty A.G. (2023). Combination of Platelet-Rich Plasma and Hyaluronic Acid vs. Platelet-Rich Plasma Alone for Treatment of Knee Osteoarthritis. Biomedicines.

[2] Gupta A. (2022). Platelet-Rich Plasma One Week Prior to Hyaluronic Acid vs. Platelet-Rich Plasma Alone for the Treatment of Knee Osteoarthritis. Biomedicines.

[3] Gupta A., Jeyaraman M., Maffulli N. (2022). Common Medications Which Should Be Stopped Prior to Platelet-Rich Plasma Injection. Biomedicines.

[4] Gupta A. (2022). Allogenic Amniotic Tissue for Treatment of Knee and Hip Osteoarthritis. Pharmaceuticals.

[5] Sokolove J., Lepus C.M. (2013). Role of inflammation in the pathogenesis of osteoarthritis: Latest findings and interpretations. Ther. Adv. Musculoskelet. Dis..

[6] Little C.B., Hunter D.J. (2013). Post-traumatic osteoarthritis: From mouse models to clinical trials. Nat. Rev. Rheumatol..

[7] Loeser R.F., Goldring S.R., Scanzello C.R., Goldring M.B. (2012). Osteoarthritis: A disease of the joint as an organ. Arthritis Rheum..

[8] Bush J.R., Beier F. (2013). TGF-β and osteoarthritis—The good and the bad. Nat. Med..

[9] Aoki C.A., Borchers A.T., Li M., Flavell R.A., Bowlus C.L., Ansari A.A., Gershwin M.E. (2005). Transforming growth factor beta (TGF-beta) and autoimmunity. Autoimmun. Rev..

[10] Gupta A. (2022). Amniotic Suspension Allograft for Treatment of Knee Osteoarthritis. Biomedicines.

[11] Gupta A., Maffulli N. (2022). Allogenic Umbilical Cord Tissue for Treatment of Knee Osteoarthritis. Sports Med. Arthrosc. Rev..

[12] Gupta A., Potty A.G., Maffulli N. (2023). Allogenic Platelet-rich Plasma for treatment of knee and hip osteoarthritis. Front. Pain. Res..

[13] Veronesi F., Giavaresi G., Tschon M., Borsari V., Nicoli Aldini N., Fini M. (2013). Clinical use of bone marrow, bone marrow concentrate, and expanded bone marrow mesenchymal stem cells in cartilage disease. Stem Cells Dev..

[14] Vangsness C.T., Farr J., Boyd J., Dellaero D.T., Mills C.R., LeRoux-Williams M. (2014). Adult human mesenchymal stem cells delivered via intra-articular injection to the knee following partial medial meniscectomy: A randomized, double-blind, controlled study. J. Bone Jt. Surg. Am..

[15] Vega A., Martín-Ferrero M.A., Del Canto F., Alberca M., García V., Munar A., Orozco L., Soler R., Fuertes J.J., Huguet M. (2015). Treatment of knee osteoarthritis with allogeneic bone marrow mesenchymal stem cells: A randomized controlled trial. Transplantation.

[16] Gupta P.K., Das A.K., Chullikana A., Majumdar A.S. (2012). Mesenchymal stem cells for cartilage repair in osteoarthritis. Stem Cell Res. Ther..

[17] Koh Y.-G., Jo S.-B., Kwon O.-R., Suh D.-S., Lee S.-W., Park S.-H., Choi Y.-J. (2013). Mesenchymal stem cell injections improve symptoms of knee osteoarthritis. Arthroscopy.

[18] Jo C.H., Lee Y.G., Shin W.H., Kim H., Chai J.W., Jeong E.C., Kim J.E., Shim H., Shin J.S., Shin I.S. (2014). Intra-articular injection of mesenchymal stem cells for the treatment of osteoarthritis of the knee: A proof-of-concept clinical trial. Stem. Cells Dayt. Ohio..

[19] Emadedin M., Aghdami N., Taghiyar L., Fazeli R., Moghadasali R., Jahangir S., Farjad R., Eslaminejad M.B. (2012). Intra-articular injection of autologous mesenchymal stem cells in six patients with knee osteoarthritis. Arch. Iran. Med..

[20] Gupta P.K., Chullikana A., Rengasamy M., Shetty N., Pandey V., Agarwal V., Wagh S.Y., Vellotare P.K., Damodaran D., Viswanathan P. (2016). Efficacy and safety of adult human bone marrow-derived, cultured, pooled, allogeneic mesenchymal stromal cells (Stempeucel^®^): Preclinical and clinical trial in osteoarthritis of the knee joint. Arthritis Res. Ther..

[21] Gupta P.K., Maheshwari S., Cherian J.J., Goni V., Sharma A.K., Tripathy S.K., Talari K., Pandey V., Sancheti P.K., Singh S. (2023). Efficacy and Safety of Stempeucel in Osteoarthritis of the Knee: A Phase 3 Randomized, Double-Blind, Multicenter, Placebo-Controlled Study. Am. J. Sports Med..

